# Genre analysis of introduction section in electrical engineering undergraduate laboratory reports

**DOI:** 10.12688/f1000research.73461.1

**Published:** 2022-02-09

**Authors:** Veeramuthu Veerappan, Mokhtarrudin Ahmad, Mohd Afizal Aris, Wei Hui Suan

**Affiliations:** 1 Faculty of Applied Communication, Multimedia University, Cyberjaya, Selangor, 63100, Malaysia; 2 City University, Selangor, Malaysia

**Keywords:** Introduction section, laboratory reports, move analysis, engineering discourse

## Abstract

Background

This study examines the genre of Engineering Laboratory Reports (ELR) introduction section written by Electrical Engineering Undergraduates in a higher learning institution. The aims of this study are to identify the rhetorical moves and combinations of move patterns used by engineering students to write introduction section of ELR.

Methods

A genre analysis was conducted to identify writing patterns and convention practices of engineering undergraduate students thus a corpus of N= 35 was selected from electrical engineering students in their final year of study. This study adopted Genre Theory as its theoretical framework, [1] analytical framework and [2] BCU approach for analysis procedure. A pilot test was conducted to determine the model that fits the best to describe moves and steps of ELR. The study benchmarks a move or step to be present in at least 60% of the reports.

Results

The finding shows the introduction consists of one main move which is providing background information of the experiment and followed by four subsequent steps which are reference to research purposes, reference to theoretical knowledge in the field, providing an overview of the study and identification of main research apparatus. The move 1 and all four steps identified above are viewed as conventional move and steps of introduction section only among undergraduates in academic context. The exemplification of finding pave ways to address grey areas of improvement in scientific written genre among laboratory instructors and academics. The method employed in this study may be replicated to analyse other sections of scientific and technical reports such as method, result, discussion and conclusion (MRDC).

Conclusions

This study posits the importance of collaboration between English for Academic (EAP) practitioners such as English-writing instructors and discipline specific specialist from engineering field to further improve on genre-based writing instruction, and to identify student learning needs.

## Introduction

Among the many academic genres, writing an investigative article has grown a lot of curiosity and thoughtfulness among the academicians and researchers. ^3^ has introduced his “move analysis” to examine the structure of introduction section in the engineering research articles. In his 1990 research, Swales suggested a “3-move schema” in writing introductions for any articles which is known as the “Create A Research Space” (CARS) model. This study contributes to a better understanding among practitioners in this discourse community on how to write effective research articles but not much study has been conducted to analyze undergraduate engineering laboratory reports. ^4^ in his paper, “ *Writing a Laboratory Report for Senior Electrical Engineering Courses: Guidelines and Recommendations,*” mentioned that in engineering pedagogics, specifically in the electrical engineering courses, laboratory report can be use as the measurement to evaluate the understanding of the theory taught in the classes.

The primary job of any scientific Introduction is to establish the purpose for doing the experiment that is to be reported. Thus, the introduction and the theoretical background were usually combined into one introductory section depending on the length and complexity of the report. ^4^ stated that the introduction creates the reader’s overall understanding of the rest of the report. He mentioned that students should only write a maximum of two A4 papers to avoid any irrelevant information being mentioned and stated in the introductory paragraph. Moreover, the introductory paragraph should also make a reference to the appropriate theory and the importance of the previous studies whenever necessary. Not all undergraduate students have experienced writing ELRs in high schools or before varsity admission. ^5^

The ability to write an effective introductory paragraph and abstract or summary will assist the writing process, as these sections, especially the introduction contains a synopsis of the whole report. According to ^6^ the inquiry based ELRs foster questioning, designing experiments and interpreting results are essential processes to become experimental scientist. ^10^ The introduction or the introductory paragraph is one of the central sections of a laboratory report. To have the readers have a better understanding and a clear guideline of the report, the introduction should also provide relevant background information and puts the study into context of the depth and challenges of an experiment. ^11^ Additionally, the introduction should include a brief overview of relevant and latest publications in the respective field.

According to the university’s guideline, an introduction of an Engineering Laboratory Report (ELR) should consist of an overview of the topic under experiment, a clear statement of purpose, the reasons to initiate the experiment, as well as general content to assist reader’s understanding into subject matter. The engineering faculty also requires students to discuss underpinning theory that leads to experiment, a short literature review on the theory, questions and even ambiguities which arose from the chosen theory. The current study attempts to fill the existing gap by investigating the specific discourse features in the Introduction section used among engineering students in writing ELR at tertiary level. The purpose of this study is to investigate what are the moves and steps as well as the combination of move and step patterns used by engineering undergraduates in writing the introduction section of ELR. These aims are to be realized with the use of Genre Theory. ^3^^,^^7^ mentioned that Genre Theory has evolved from the study of discourse and linguistic analysis to further describe and explain why the members which belongs to a certain discourse community use the language the way they do. The interpretative characteristic of genre theory made it widely accepted and used in genre-based studies among academics and linguists. ^8^

## Methods

A genre analysis was conducted to determine the moves and steps that occur in the introduction section by adapting the categories outlined by Ref. 1 framework. Prior to using Ngowu’s model, a pilot test was conducted to examine the suitability of this model to the current study on introduction section of ELR. 15 ELRs were selected and examined to determine the match in description. There were more than 60% match in the categories outlined by the model and ELRs under examination, making it the most suitable analytical framework that can be modified and replicated. The total sample size collected was N=74. These samples were further minimized and scrutinized to select the ELRs that contain most complete and comprehensive information. Quality of data, amount of information, nature of topic, scope of study, design and method used in a qualitative study are the few factors used in determining sample size. ^9^ In this study, the sample was based on selected ELR’s that achieved a score of 4 marks out of 5 marks. The sample size of this study is maintained at N=35 as these samples best represented the electrical engineering laboratory genre and reached a saturation level with similar recurring moves and steps. To further validate the data, semi-structured interview was conducted with an engineering content specialist. The total intake of electrical engineering students in this tertiary institution is not more than 100 students a year and, in each trimester, these students are engaged in writing at least 4 ELRs. The data consist of ELRs that have obtained a higher rating of at least 4 out of 5 marks. The procedures for conducting move analysis in this study adapted a corpus-based model outlined by Ref. 2 BCU approach.

As to address the trustworthiness of this study, a coding protocol was developed with definitions and examples for mandatory, conventional and optional moves and an inter-rater reliability check was conducted among three coders, consisting firstly the researcher, secondly an engineering content specialist and thirdly a language expert to determine disagreement or discrepancies and the coding protocol was revised. This coding scheme assists in identification of introduction section and also controls the variability in the analysis, which guides other coders apart from the researcher himself to identify the moves and steps. Some textbooks and research articles were also used as a guide such as the textbooks written by Refs. 8,  9 on engineering and technical writing and research articles reporting engineering writing, ^12^^–^^14^ and genre-based studies of written discourse in other engineering disciplines. ^15^^–^^17^ There were three coders involved in the development and modification of the coding scheme who is the researcher himself, secondly an engineering content specialist and thirdly a language instructor. The reason of including two other coders apart from the researcher is to ensure inter-coder reliability. The second and third coders were trained to read two similar samples of ELR’s and identify the moves and steps in the Introduction section.

Ethical Approval Number: EA1582021 approved by Technology Transfer Office, Multimedia University.

## Results and discussion

In order to depict how the texts are analysed, the extracts of text from ELR corpus which show moves and steps are exemplified. The move and steps are identified such as (M1S1 means Move 1 Step 1). The extracted text used for exemplification is bolded and italicised to show the differences in analysis. The analysis shows that the introduction consists of one main move which is providing background information of the experiment and followed by three subsequent steps which are reference to research purposes, reference to theoretical knowledge in the field, providing an overview of the study and identification of main research apparatus. This finding has more than 80% consistency to the findings of a previous studies by Refs. 17, 18 on biochemistry articles.

### Move 1: Presenting background information

Move 1 of the ELR is written to present background information of the experiments consists of 4 steps. Firstly, Move 1 Step 1 the reference to research purposes. This statement is written prior to all other information as to guide the writer throughout the reporting process of laboratory experiment. All the reports have stated at least two objectives that it aimed to achieve in the experiment. Move 1 Step 2 provides an overview of the topic under study. This step is important to give readers general information about the experiment. Move 1 Step 3 provides the theoretical knowledge of the field under study. Move 1 Step 4 is identification of main research apparatus. This step mainly outlines the apparatus used in the current experiment. It was observed that in Move 1 Step 4, most of the ELRs did not provide detailed description of the apparatus used.

### Move 1 Step 1: Reference to research purposes

The Move 1 Step 1, reference to research purposes is written to state the objectives of the initial experiment. This move can be considered the most important move in the introduction section to inform readers the main aims of conducting the experiment. The completed report can be understood by readers if the objectives are clearly stated before moving on to other steps in the introduction section. Based on the analysis of 35 ELR’s compiled, this step occurred as Move 1 Step 1 in 24 ELR’s or 69% from the total percentage. This is a significant finding as it is representative to the overall moves in introduction section.

### Move 1 Step 2: Giving an overview of the topic under study

Move 1 Step 2 provides an overview of the topic under study. It gives general information about what the experiment is all about, states the characteristics of the variables under study, the main terms used in this experiment, short definition of the functions of each variables and features used in the experiment that gives an overall view of the experiment which has been conducted. The analysis reveal that this move has occurred in 24 reports or 69% of the total report. This has made this move conventional in the introduction section based on occurrence rate. Although this step frequently occurs in the reports, yet its sequence is not in accordance. It was observed that this step has occurred only in 9 reports as step 2. This shows that the move pattern in introduction section is not always in sequence of M1S1-M1S2-M1S3-M1S4.

### Move 1 Step 3: Reference to theoretical knowledge in the field.

Move 1 Step 3 provides reference to the theoretical knowledge in the field. This move provides students or readers with sufficient mathematical or theoretical background to understand how the experiment works, what has the earlier assumptions indicated and how the experiment is related to the theoretical knowledge. This section may be written in short, if it can be well understood and connection can be made with the measurement of an experiment. Based on the analysis on 35 ELR’s, this step occurred in 21 reports, which totals up to 60% of occurrence from the overall reports. This finding is significant, making this step as a conventional step in writing introduction section. However, the analysis also shows that this step occurred as step 3 in Move 1 in 17 instances or 49% only.

### Move 1 Step 4: Stating the main apparatus used to conduct the experiment

The Move 1 Step 4 is written to state the main apparatus used to conduct the experiment. In this step, all the equipment used are usually outlined and very small number of ELR’s provide detailed description of each apparatus used for the experiment. Based on the analysis of 35 ELR’s compiled, this step occurred in the introduction section of 33 ELR’s or 94% of the total reports. This is a significant finding as it is representative to the overall moves in introduction section. However, another finding shows that this step has occurred as Move 1 Step 4 in only 17 ELR’s or 49% only. Hence, this indicates that the identification of main apparatus has occurred as other steps in move 1. This shows that not all steps in ELR’s occur in a pre-determined sequential step. The analysis also indicates that Move 1 Step 1 was not written in 3 ELR’s.

### Combination of move patterns in Introduction section

In this section, the analysis of ELR’s focuses to determine the sequence of move and steps with which steps to begin and end the introduction section. As noted in previous analysis, the length of each steps varies with Move 1 Step 1 that states the objectives of the study to be the shortest of all 4 steps in move 1. In this step, students use action verbs to state the objectives without detailed elaboration. This step clearly states the aims to be achieved by the end of an experiment. It is clear that 24 ELR’s or 69% of the reports began with the statement of objectives. Although most of the reports starts with M1S1, this finding cannot be generalized to overall ELR’s as some start from M1S1. 7 ELR’s or 20 % of the overall reports starts with M1S2, 2 ELR’s or less than 6% of the reports start with M1S3 and only 1 report or 3% starts from M1S4 and 17 ELR’s or 49% of the total reports end with M1S1. This is the last step in presenting background information of the experiment. This step is frequently adopted to end the introduction section and before moving to method section. Next, 9 ELR’s or 26% of the reports end with M1 S3, while 6 ELR’s or 17% of the reports end with M1S2 and only 3 report ends with M1S1. Based on the occurrence of each steps in Move 1 of introduction section, move 1 and all three steps identified and discussed above are viewed as mandatory and conventional steps of introduction section. The model proposed in Table 1 below encapsulates the Move and Steps made by undergraduate students of Electrical Engineering in writing their laboratory reports.

**Table 1.** Model for Introduction section of ELR

Move 1	Presenting background information
by Move 1 Step 1	Reference to research purpose
by Move 1 Step 2	Providing an overview of the study
by Move 1 Step 3	Reference to theoretical knowledge in the field
by Move 1 Step 4	Identification of main research apparatus

## Conclusion

There are several conclusions that can be drawn from this study. Mismatches between the guidelines set by the university and the final output of students are identified, firstly, the omission of background theories or pertinent literature review. The historical background of previous studies is important as it can be replicated and used for the current experiment. It is also to convince the readers the theory is viable to be used in the experiment. Secondly, it is lacking in the content to orientate the readers with enough general ideas into the subject of experiment. Thirdly, although it is the most applicable approach to enforce students experiential learning, this may not contribute or add on to the body of knowledge in engineering field. This study propose collaboration between English for Academic (EAP) practitioners such as English writing instructors and discipline specific specialist from engineering field to further improve on genre-based writing instruction to identify students’ learning needs. The limitation of this study is it focuses only on ELR written in electrical engineering domain and findings cannot generalized to other sub-disciplines and the omission of data triangulation from various sources such as interviewing and expert validation which could contribute to better understanding of why a particular discourse community writes the text the way they do.

## Data availability

Figshare. Genre Analysis of introduction section in electrical engineering undergraduate laboratory reports. DOI: https://doi.org/10.6084/m9.figshare.14881911  ^19^

Data are available under the terms of the Creative Commons Zero “No rights reserved” data waiver (CC BY 4.0 Public domain dedication).
