# Genre analysis of introduction section in electrical engineering undergraduate laboratory reports

**DOI:** 10.12688/f1000research.73461.2

**Published:** 2023-04-27

**Authors:** Veeramuthu Veerappan, Mokhtarrudin Ahmad, Kavitha Balakrishnan, Mohd Afizal Aris, Wei Hui Suan

**Affiliations:** 1 Learning Institute for Empowerment, Multimedia University, Cyberjaya, Selangor, 63100, Malaysia; 2 Faculty of Applied Communication, Multimedia University, Cyberjaya, Selangor, 63100, Malaysia; 3 City University, Selangor, Malaysia

**Keywords:** Introduction section, laboratory reports, move analysis, engineering discourse

## Abstract

Background

This study examines the genre of Engineering Laboratory Reports (ELR) introduction section written by Electrical Engineering Undergraduates in a higher learning institution. The aims of this study are to identify the rhetorical moves and combinations of move patterns used by engineering students to write introduction section of ELR.

Methods

A genre analysis was conducted to identify writing patterns and convention practices of engineering undergraduate students thus a corpus of N= 35 was selected from electrical engineering students in their final year of study. This study adopted Genre Theory as its theoretical framework, [1] analytical framework and [2] BCU approach for analysis procedure. A pilot test was conducted to determine the model that fits the best to describe moves and steps of ELR. The study benchmarks a move or step to be present in at least 60% of the reports.

Results

The finding shows the introduction consists of one main move which is providing background information of the experiment and followed by four subsequent steps which are reference to research purposes, reference to theoretical knowledge in the field, providing an overview of the study and identification of main research apparatus. The move 1 and all four steps identified above are viewed as conventional move and steps of introduction section only among undergraduates in academic context. The exemplification of finding pave ways to address grey areas of improvement in scientific written genre among laboratory instructors and academics. The method employed in this study may be replicated to analyse other sections of scientific and technical reports such as method, result, discussion and conclusion (MRDC).

Conclusions

This study posits the importance of collaboration between English for Academic (EAP) practitioners such as English-writing instructors and discipline specific specialist from engineering field to further improve on genre-based writing instruction, and to identify student learning needs.

## Introduction

Among the many academic genres, writing an investigative article has grown a lot of curiosity and thoughtfulness among the academicians and researchers ^1^ has introduced his “move analysis” to examine the structure of introduction section in the engineering research articles. ^2^ This study contributes to a better understanding among practitioners in this discourse community on how to write effective research articles but not much study has been conducted to analyse undergraduate engineering laboratory reports. ^3^ In his paper, “ *Writing a Laboratory Report for Senior Electrical Engineering Courses: Guidelines and Recommendations,*” mentioned that in engineering pedagogics, specifically in the electrical engineering courses, laboratory report can be use as the measurement to evaluate the understanding of the theory taught in the classes.

The primary job of any scientific introduction is to establish the purpose for doing the experiment that is to be reported. Thus, the introduction and the theoretical background were usually combined into one introductory section depending on the length and complexity of the report. ^3^ stated that the introduction creates the reader’s overall understanding of the rest of the report. He mentioned that students should only write a maximum of two A4 papers to avoid any irrelevant information being mentioned and stated in the introductory paragraph. Moreover, the introductory paragraph should also make a reference to the appropriate theory and the importance of the previous studies whenever necessary. Not all undergraduate students have experienced writing ELRs in high schools or before varsity admission. ^4^

The ability to write an effective introductory paragraph and abstract or summary will assist the writing process, as these sections, especially the introduction contains a synopsis of the whole report. According to Ref. 5 the inquiry based ELRs foster questioning, designing experiments and interpreting results are essential processes to become experimental scientist. ^6^ The introduction or the introductory paragraph is one of the central sections of a laboratory report. To have the readers have a better understanding and a clear guideline of the report, the introduction should also provide relevant background information and puts the study into context of the depth and challenges of an experiment. ^7^ Additionally, the introduction should include a brief overview of relevant and latest publications in the respective field.

According to the university under study’s guideline, an introduction of an Engineering Laboratory Report (ELR) should consist of an overview of the topic under experiment, a clear statement of purpose, the reasons to initiate the experiment, as well as general content to assist reader’s understanding into subject matter. The engineering faculty also requires students to discuss underpinning theory that leads to experiments, a short literature review on the theory, questions and even ambiguities which arose from the chosen theory. The current study attempts to fill the existing gap by investigating the specific discourse features in the Introduction section used among engineering students in writing ELR at tertiary level. Previous studies in similar field have focused on underpinning theories, rather than tertiary driven experiments. The focus is not explicitly on laboratory work, but on inquiry as cited by Refs. 8– 12; and practical work, ^12^ hands-on practices. ^13^ The purpose of this study is to investigate what are the moves and steps as well as the combination of move and step patterns used by engineering undergraduates in writing the introduction section of ELR. These aims are to be realized with the use of Genre Theory. ^1^^,^^14^ mentioned that Genre Theory has evolved from the study of discourse and linguistic analysis to further describe and explain why the members which belongs to a certain discourse community use the language the way they do. The interpretative characteristic of genre theory made it widely accepted and used in genre-based studies among academics and linguists. ^15^

## Methods

A genre analysis was conducted to determine the moves and steps that occur in the introduction section by adapting the categories outlined by Ref. 2 framework. Prior to using Ngowu’s model, a pilot test was conducted to examine the suitability of this model to the current study on introduction section of ELR. 15 ELRs were selected and examined to determine the match in description. There were more than 60% match in the categories outlined by the model and ELRs under examination, making it the most suitable analytical framework that can be modified and replicated. The total sample size collected was N=74. These samples were further minimized and scrutinized to select the ELRs that contain most complete and comprehensive information. Quality of data, amount of information, nature of topic, scope of study, design and method used in a qualitative study are the few factors used in determining sample size. ^9^ In this study, the sample was based on selected ELR’s that achieved a score of 4 marks out of 5 marks. The sample size of this study is maintained at N=35 as these samples best represented the electrical engineering laboratory genre and reached a saturation level with similar recurring moves and steps. To further validate the data, semi-structured interview was conducted with an engineering content specialist. The total intake of electrical engineering students in this tertiary institution is not more than 100 students a year and, in each trimester, these students are engaged in writing at least 4 ELRs. The data consist of ELRs that have obtained a higher rating of at least 4 out of 5 marks. The procedures for conducting move analysis in this study adapted a corpus-based model outlined by Ref. 16 BCU approach.

As to address the trustworthiness of this study, a coding protocol was developed with definitions and examples for mandatory, conventional and optional moves and an inter-rater reliability check was conducted among three coders, consisting firstly the researcher, secondly an engineering content specialist and thirdly a language expert to determine disagreement or discrepancies and the coding protocol was revised. This coding scheme assists in identification of introduction section and also controls the variability in the analysis, which guides other coders apart from the researcher himself to identify the moves and steps. Some textbooks and research articles were also used as a guide such as the textbooks written by Refs. 15, 17 on engineering and technical writing and research articles reporting engineering writing, ^18^^–^^20^ and genre-based studies of written discourse in other engineering disciplines. ^21^^–^^23^ There were three coders involved in the development and modification of the coding scheme who is the researcher himself, secondly an engineering content specialist and thirdly a language instructor to ensure inter-coder reliability. The second and third coders were trained to read two similar samples of ELR’s and identify the moves and steps in the Introduction section.

Ethical Approval Number: EA1582021 approved by Technology Transfer Office, Multimedia University.

## Results and discussion

In order to depict how the texts are analysed, the extracts of text from ELR corpus which show moves and steps are exemplified. The move and steps are identified such as (M1S1 means Move 1 Step 1). The extracted text used for exemplification is bolded and italicised to show the differences in analysis. The analysis shows that the introduction consists of one main move which is providing background information of the experiment and followed by three subsequent steps which are reference to research purposes, reference to theoretical knowledge in the field, providing an overview of the study and identification of main research apparatus. This finding has more than 80% consistency to the findings of a previous studies by Refs. 23, 24 on biochemistry articles.

Move 1: Presenting background information occurred in all 35 ELRs or 100% (Obligatory) shows this move as the most important element in ELR introduction. This statement is written prior to all other information as to guide the writer throughout the reporting process of laboratory experiment and is important to give readers general information about the experiment. This move consists of 4 steps. Firstly, move 1 step 1 the reference to research purposes, secondly, move 1 step 2 provides an overview of the topic under study, thirdly, move 1 step 3 provides the theoretical knowledge of the field under study and finally move 1 step 4 identification of main research apparatus. Move 1: Presenting background information was written in all 35 ELRs or 100% (Obligatory) that makes this move as the most important structure in ELR introduction. This move is significant as it represents the overall moves in the introduction section.

Move 1 Step 1, reference to research purposes is written to state the objectives of the initial experiment. This step is considered as the most important step in introduction section that serves to inform readers the aims of conducting laboratory experiment. The completed report can only be understood by readers if the objectives are clearly stated before moving on to other steps. Based on the analysis of 35 ELR’s compiled, this step occurred as Move 1 Step 1 in 24 ELR’s or 69% (conventional). This supports the notion that there is a gap in stating the research objectives clearly and this hinder reader’s understanding the purpose of the conducting and reporting experiments.

Move 1 Step 2 provides an overview of the topic under study. It gives general information about what the experiment is all about, states the characteristics of the variables under study, the main terms used in this experiment, short definition of the functions of each variable and features used that gives an overall view of the experiment conducted. This step has occurred in 33 reports or 94% of the total report (conventional), however, it was in correct sequence in only 9 reports as step 2. This shows that the move pattern in introduction section is not always in sequence of M1S1-M1S2-M1S3-M1S4.

Move 1 Step 3 provides reference to the theoretical knowledge in the field. This move provides students or readers with sufficient mathematical or theoretical background to understand how the experiment works, what has the earlier assumptions indicated and how the experiment is related to the theoretical knowledge. This section may be written in short if it can be well understood and connection can be made with the measurement of an experiment. Move 1 Step 3 occurred in only 20 reports or 57% (optional). These reports are lacking in referencing underpinning theories and citing previous literature thus making the report less concrete. Moreover, this step occurred as step 4 in 17 instances or 49% thus neither in accordance with university guidelines nor in correct sequence of M1S1-M1S2-M1S3 and M1S4.

Move 1 Step 4: Stating the main apparatus used to conduct the experiment occurred in the introduction section of 33 ELR’s or 94% (conventional) but 29 reports or 83% of the total reports just stated or outlined the main apparatus used without detailed explanation on how to use it. This is another lack identified that most of the ELRs did not provide detailed description of the apparatus used. Moreover, this step has occurred as step 4 in only 17 ELR’s or 49% only. It can be inferred lack of compliance among undergraduates reporting ELR’s in sequential orders outlined by the university. This also shows lack of clarity in describing what are the apparatus used, why it is used and how this equipment helps in the experiment process.

The analysis to the combination of move patterns focuses to determine the sequence of move and steps used to begin and end the introduction. As noted in previous analysis, the length of each step varies with M1S1 that shows the objectives of the experiment written the shortest of all 4 steps. In this step, students use action verbs to state the objectives without detailed elaboration. This step clearly states the aims to be achieved by the end of experiment. 24 ELRs or 69% start with M1S1, 7 ELR’s or 20% of the reports starts with M1S2, 2 ELR’s or less than 6% of the reports start with M1S3 and only 1 report or 3% starts from M1S4 and 17 ELR’s or 49% of the reports end with M1S1. This step is frequently adopted to end the introduction section and before moving to method section. Next, 9 ELR’s or 26% of the reports end with M1S3, while 6 ELR’s or 17% of the reports end with M1S2 and only 3 report ends with M1S1. The occurrence of move 1 and all three steps identified and discussed above are reported as obligatory, conventional and optional. The combination of move and steps show most of the steps overlap against each other and not in sequential order. This may pose challenges to laboratory instructors and supervisors to comprehend and assess the reports with clarity as only moderate level of compliance is shown by undergraduates to write the reports in the format and conventions outlined by the university. The model proposed in Table 1 below encapsulates the move and steps made by undergraduate students of Electrical Engineering in writing their laboratory reports.

**Table 1.** Model for Introduction section of ELR.

Move 1	Presenting background information
by Move 1 Step 1	Reference to research purpose
by Move 1 Step 2	Providing an overview of the study
by Move 1 Step 3	Reference to theoretical knowledge in the field
by Move 1 Step 4	Identification of main research apparatus

## Conclusion

There are several conclusions that can be drawn from this study. When the findings are compared against the university guidelines, few mismatches are identified. Firstly, the omission of background theories and pertinent literature review. Minimal discussion of previous studies or replication shows undergraduates are taking the laboratory experiments to their own hands. It is also hard to convince or gain wider audience when the experiment is conducted without stating a viable theoretical background. Secondly, it is lacking in content to orientate the readers with sufficient general information about the subject of experiment and the reasons for using certain apparatus was also not briefly explained. Thirdly, a proportion of findings reveal not much attention was paid to writing the report in sequential orders. Lastly, although laboratory report writing is the most applicable genre to enforce students experiential learning, it may not contribute or add to new literature or body of knowledge if proper referencing and citations are not practiced. This study recommends collaboration between English for Academic (EAP) practitioners such as English writing instructors and discipline specific specialist from engineering field to further improve on genre-based writing instruction to support students in comprehending the requirements of the ELR genre. This study is limited to ELR’s written in electrical engineering domain and the absence of analysis on its linguistic features, hence the findings cannot be generalized to other sub-disciplines in engineering. This study proposes data triangulation from various sources such as interviews and expert validation for future research that could yield more comprehensive findings to understand why a particular discourse community writes the text the way they do.

## Data availability

Figshare. Genre Analysis of introduction section in electrical engineering undergraduate laboratory reports. DOI: https://doi.org/10.6084/m9.figshare.14881911.v3. ^25^

Data are available under the terms of the Creative Commons Zero “No rights reserved” data waiver (CC BY 4.0 Public domain dedication).
