# Genre analysis of introduction section in electrical engineering undergraduate laboratory reports

**DOI:** 10.12688/f1000research.73461.3

**Published:** 2024-03-22

**Authors:** Veeramuthu Veerappan, Mokhtarrudin Ahmad, Kavitha Balakrishnan, Mohd Afizal Aris, Wei Hui Suan

**Affiliations:** 1 Learning Institute for Empowerment, Multimedia University, 63100 Cyberjaya, Selangor, Malaysia; 2 Faculty of Applied Communication, Multimedia University, Cyberjaya, Selangor, 63100, Malaysia; 3 City University, Selangor, Malaysia

**Keywords:** Introduction section, laboratory reports, move analysis, engineering discourse

## Abstract

**Background:**

This study examines the genre of Engineering Laboratory Reports (ELR) introduction section written by Electrical Engineering Undergraduates in a higher learning institution. The aims of this study are to identify the rhetorical moves and combinations of move patterns used by electrical engineering (EE) students to write introduction section.

**Method:**

A genre analysis was conducted to identify writing patterns and convention practices of engineering undergraduate students thus a corpus of N = 35 was selected from electrical engineering students in their final year of study. This study adopted Genre Theory as its theoretical framework, Ngowu 1997 analytical framework and BCU approach for analysis procedure. A pilot test was conducted to determine the model that fits the best to describe moves and steps of ELR. Coding scheme was developed and intercoder reliability showed a significance of 0.91 The study benchmarks a move or step to be present in at least 60% of the reports.

**Results:**

The finding shows the introduction consists of one main move which is providing background information of the experiment and followed by four subsequent steps which are reference to research purposes, reference to theoretical knowledge in the field, providing an overview of the study and identification of main research apparatus. The move 1 and all four steps identified above are viewed as obligatory, conventional and optional move and steps in introduction section among undergraduates in academic context. The exemplification of finding shows lack of compliance among undergraduates to produce EELR based on university's guideline in discussing previous literature and underpinning theories, lack of referencing and citation, absence in describing apparatus used and non-sequential moves steps.

**Conclusion:**

This study posits the importance of collaboration between English for Academic (EAP) practitioners such as English-writing instructors and discipline specific specialist from engineering field to further improve on genre-based writing instruction, and to identify student learning needs. The method employed in this study may be replicated to analyse other sections of scientific and technical reports such as method, result, discussion and conclusion (MRDC) that may pave ways to address grey areas for improvement in this genre.

## Introduction

Among the many academic genres, writing an investigative article has grown a lot of curiosity and thoughtfulness among the academicians and researchers. ^1^ Has introduced his “move analysis” to examine the structure of introduction section in the engineering research articles. ^2^ This study contributes to a better understanding among practitioners in this discourse community on how to write effective research articles but not much study has been conducted to analyze electrical engineering laboratory reports (EELR). ^3^ In his paper, “ *Writing a Laboratory Report for Senior Electrical Engineering Courses: Guidelines and Recommendations,*” mentioned that in engineering pedagogics, specifically in the electrical engineering courses, laboratory report can be use as the measurement to evaluate the understanding of the theory taught in the classes.

Research indicates that tertiary level engineering programs are challenging for both learners and educators. This can be seen with high dropout rates, low academic achievement and lack of diversity within academic programs. ^4^^–^^7^

Across the globe, governments, corporates, and scholars are widely acknowledging the need for reform in tertiary engineering education. ^8^ Substantial efforts and resources have been funded to improve the quantity and quality of engineering graduates. However, despite these tantamount investments, there has been limited improvement and reformation in this field of education. ^9^

The attention given to the actions and behaviours of teachers, students, administrators and institutions have often overshadowed the gist and essence of engineering knowledge. This study aims to address the rhetorical patterns, structure and conventions of EELR writing among EE students in a higher learning institution. The EE education specializes in scientific foundation, by emphasising graduates to gain expertise in electrical field. It encompasses elements of traditional EE curriculum such as circuit building and electricity transmission, alternative energy, and high current transfer. ^10^

Beck and Young (2005) ^11^ contend that regional context shapes professional identity and their specialised knowledge while Bernstein ^12^ identified the connection between professional identity and knowledge through “inner dedication” to foster awareness and dedication among EE learners and educators. The cutting-edge engineering education are shifting its understanding or epistemology by moving from foundational sciences to applied sciences, from theoretical knowledge to practical problem solving and design skills, and from academic realm to professional world. New perspectives and methodologies are introduced within disciplines like EE. Academics at universities are becoming increasingly cognizant to these epistemological changes and recognise that successful navigation through these stages are crucial for students’ achievement. ^4^ Thus, an engineering project can serve as a distinctive teaching pedagogy within EE programs that entails hands-on laboratory work and the creation of engineering artifacts. ^13^

The primary job of any scientific introduction is to establish the purpose for doing the experiment that is to be reported. Thus, the introduction and the theoretical background were usually combined into one introductory section depending on the length and complexity of the report. ^3^ stated that the introduction creates the reader’s overall understanding of the rest of the report. He mentioned that students should only write a maximum of two A4 papers to avoid any irrelevant information being mentioned and stated in the introductory paragraph. Moreover, the introductory paragraph should also make a reference to the appropriate theory and the importance of the previous studies whenever necessary. Not all undergraduate students have experienced writing ELRs in high schools or before varsity admission. ^14^

The ability to write an effective introductory paragraph and abstract or summary will assist the writing process, as these sections, especially the introduction contains a synopsis of the whole report. According to Ref. ^15^ the inquiry based ELRs foster questioning, designing experiments and interpreting results are essential processes to become experimental scientist. ^16^ The introduction or the introductory paragraph is one of the central sections of a laboratory report. To have the readers have a better understanding and a clear guideline of the report, the introduction should also provide relevant background information and puts the study into context of the depth and challenges of an experiment. ^17^ Additionally, the introduction should include a brief overview of relevant and latest publications in the respective field.

Based on the university guidelines, below are the rhetorical structures required for the introduction section. 1. This section is to discuss the theoretical aspects leading to the experiment. 2. Typically, this involves the historical background of the theories published in the research literature and the questions or ambiguities arose in this theoretical work. 3. Citations for the sources of information should be given in one of the standard bibliographic formats (for example, using square brackets with the corresponding number [2] that points to the List of References). 4. Explore this background to prepare the readers to read the main body of the report. 5. It should contain sufficient materials to enable the readers to understand why the set of data are collected, and what are the salient features to observe in the graph, charts and tables presented in the later sections. 6. Depending on the length and complexity of the report, the introduction and the theoretical background may be combined into one introductory section (Faculty of Engineering).

According to the university under study’s guideline, an introduction of an Engineering Laboratory Report (ELR) should consist of an overview of the topic under experiment, a clear statement of purpose, the reasons to initiate the experiment, as well as general content to assist reader’s understanding into subject matter. The engineering faculty also requires students to discuss underpinning theory that leads to experiments, a short literature review on the theory, questions and even ambiguities which arose from the chosen theory. The current study attempts to fill the existing gap by investigating the lacks in the Introduction section against the guidelines provided by the university. Previous studies in similar field have focused on underpinning theories, rather than tertiary driven experiments. The focus is not explicitly on laboratory work, but on inquiry as cited by Refs. 18– 22 and practical work, ^22^ hands-on practices. ^23^ The purpose of this study is to investigate what are the moves and steps as well as the combination of move and step patterns used by engineering undergraduates in writing the introduction section of ELR. These aims are to be realized with the use of Genre Theory. ^1^^,^^24^ mentioned that Genre Theory has evolved from the study of discourse and linguistic analysis to further describe and explain why the members which belongs to a certain discourse community use the language the way they do. The interpretative characteristic of genre theory made it widely accepted and used in genre-based studies among academics and linguists. ^25^

## Methods

The study descriptively examines EELRs, focusing on analyzing rhetorical moves and steps. It utilizes a qualitative research approach to explore and elucidate the various moves and steps present in EELRs. Descriptive research is chosen to portray the natural state of people, events, and conditions whereby Information is sourced from physical settings, records, documents, objects, materials, and individuals directly associated with the EELRs. ^26^ A genre analysis was conducted to determine the moves and steps that occur in the introduction section by adapting the categories outlined by Ref. 2 framework. Prior to using Ngowu’s model, a pilot test was conducted to examine the suitability of this model to the current study on introduction section of EELR.

15 EELRs were selected and examined to determine the match in the description. There were more than 60% match in the categories outlined by the model and EELRs under examination, making it the most suitable analytical framework that can be modified and replicated. The total sample size collected was N=74. These samples were further scrutinized and minimized to select the EELRs that contain most complete and comprehensive information. Quality of data, amount of information, nature of topic, scope of study, design and method used in a qualitative study are the few factors used in determining sample size. ^27^ To control the variable, only EELRs that received distinction grades of 4 and above score over maximum score of 5 awarded by lab instructors were selected to maximise the possibility that it conforms to EE disciplinary standards. The sample size of this study is maintained at N=35 as these samples best represented the EE laboratory genre and reached a saturation level with similar recurring moves and steps. The corpus consisting N=35 was converted and compiled into pdf and the number of words obtained are 47194.

To further validate the data, semi-structured interview was conducted with an engineering content specialist. According to the subject specialist, the total intake of electrical engineering students in this tertiary institution is not more than 100 students a year and, in each trimester, these students are engaged in writing at least 4 EELRs. ^28^ stated that the sample size for a qualitative study is influenced by several factors, including the quality of data, scope of the study, nature of topic, information richness from each subject, the qualitative methodology utilized, and the study’s design. Thus, the data set of N=35 represents the final year EE student population of this institution. The procedures for conducting move analysis in this study adapted a corpus-based model outlined by Ref. 29 BCU approach. This approach adopted ^30^ and followed the procedures to determine the rhetorical function and meaning of each move and step of each text, conduct a pilot-coding to test and fine-tune the definitions of move purposes manually by hand, develop a coding protocol with clear definitions and examples of move types and steps, code the full set of texts, with an inter-rater reliability check to confirm a clear understanding of move definitions and realization of moves/steps, revise the coding protocol to resolve any discrepancies revealed by the inter-rater reliability check or newly discovered’ moves/steps, and re-coding problematic areas, conduct analysis on move features and other corpus-facilitated analyses and finally describe the corpus of texts in terms of obligatory, conventional or optional move structures. As to address the trustworthiness of this study, a coding protocol was developed with definitions and examples for mandatory, conventional and optional moves and an inter-coder reliability check was conducted among three coders. A coding scheme was developed to identify rhetorical moves and steps in introduction section and also to control the variability in the analysis that guide all coders. There were three coders involved in the development and modification of the coding scheme who is the researcher himself, secondly an engineering content specialist and thirdly a language instructor. The reason for including two other coders apart from the researcher is to ensure inter-coder reliability. The second and third coders were trained to read two similar samples of EELR’s and identify the moves and steps in the Introduction section. Cohen & Kappa coefficient, a statistical measure that takes into consideration random agreement between coders, was used to evaluate intercoder reliability. To determine the degree of agreement above and beyond what would be predicted by chance, kappa statistics were computed. An online calculator was used to calculate Kappa statistics by comparing the coded data for a subset of the EELRs from all three coders. Following their separate codes for the subset of reports, the coders got together to discuss their consensus and differences. Each coder then went on to rate nine more reports. With a coefficient of 0.91, the Kappa coefficient obtained from this analysis demonstrated a high degree of agreement. This supports the validity of the study’s conclusions and shows a high level of dependability in the coding procedure.

Some textbooks and research articles were also used as a guide such as the textbooks written by Refs. 25,  27 on engineering and technical writing and research articles reporting engineering writing, ^31^^–^^33^ and genre-based studies of written discourse in other engineering disciplines. ^34^^–^^36^

Ethical Approval Number: EA1582021 approved by Technology Transfer Office, Multimedia University.

## Results and discussion

To depict how the texts are analysed, the extracts of text from EELR corpus which show moves and steps are exemplified. The move and steps are identified such as (M1S1 means Move 1 Step 1). The extracted text used for exemplification is bolded and italicised to show the differences in analysis. The analysis shows that the introduction consists of one main move which is providing background information of the experiment and followed by three subsequent steps which are reference to research purposes, reference to theoretical knowledge in the field, providing an overview of the study and identification of main research apparatus. This finding has more than 80% consistency to the findings of a previous studies by Refs. 30, 36 on biochemistry articles.

### Move 1: Presenting background information.

This move occurred in all 35 ELRs or 100% (Obligatory) shows this move as the most important element in ELR introduction. This statement is written prior to all other information as to guide the writer throughout the reporting process of laboratory experiment and is important to give readers general information about the experiment. It is written to present background information of the experiments consists of 4 steps. Firstly, Move 1 Step 1 the reference to research purposes. Move 1 Step 2 provides an overview of the topic under study. Move 1 Step 3 provides the theoretical knowledge of the field under study and Move 1 Step 4 is identification of main research apparatus. Move 1: Presenting background information was written in all 35 ELRs or 100% (Obligatory) that makes this move as the most important structure in ELR introduction. The extracted text used for exemplification is italicised to show the differences from the analysis. The following example shows how the modifications are made to the extracted text in this study. The example below is taken from the introduction section of ELR 25.

**Table 1.** Exemplification of Moves and Steps in Introduction Section extracted from ELR 25.

Move 1 Presenting Background Information Move 1 Step 1 Reference to research purposes	*To verify Thevenin’s Theorem.* *To verify Superposition Theorem.*
Move 1 Step 2 Providing an overview of the study Move 1 Step 3 Reference to theoretical knowledge in the field	*By measuring the short-circuit current I _SC_ flowing through a wire that connects X to Y, the value of R _TH_ (or Z _TH_) can be calculated as the ratio of V _TH_ over I _SC._ (Phasor values are to be used when calculating the Thevenin equivalent impedance.) (S1) The series combination of V _TH_ and R _TH_ (or Z _TH_) is the equivalent circuit of the black box. (S2) By equivalent, it means the voltage across and current through any circuit element that is connected between terminals X and Y of the black box will be the same as the case when that circuit element is connected in series with R _TH_ (or Z _TH_) and V _TH_. (S3) The theorem is valid provided that the circuit inside the “black box” is linear. (S4) The load resistor R _L_ (or load impedance Z _L_), however, may not be linear. (S5)* *Thevenin’s Theorem is a very useful and frequently used theorem in circuit analysis(S6) Consider a load resistor R _L_ (or load impedance Z _L_) that is connected to a “black box” as shown in Figure 1. (S7) The black box can contain any combination of circuit elements. (S8) Thevenin’s Theorem states that insofar as the load resistor R _L_ (or load impedance Z _L_) is concerned, the black box can be represented by a series combination of an ideal voltage source, V _TH_, and a resistor, R _TH_ (or impedance Z _TH_). V _TH_ is known as the Thevenin equivalent voltage source. (S9) Its value can be found by measuring the open-circuit voltage between terminals X and Y when the resistor R _L_ is removed. (S10) R _TH_ is called the Thevenin equivalent resistance. (S11) (Z _TH_ is called the Thevenin equivalent impedance.) (S12)*
Move 1 Step 4 Identification of main research apparatus	***“**Circuit Theory” experiment board* *DC Power Supply* *Dual-trace Oscilloscope* *Function Generator* *Digital Multimeter* *Connecting wires*

**Move 1 Step 1: Reference to research purposes**

Reference to research purposes is written to state the objectives of the initial experiment. This step is considered as the most important step in the introduction section that serves to inform readers of the aims of conducting laboratory experiments. The completed report can only be understood by readers if the objectives are clearly stated before moving on to other steps. However, based on the analysis of 35 ELR’s compiled, this step occurred as Move 1 Step 1 only in 24 ELR’s or 69% (conventional). This hinders reader’s from understanding the purpose of the conducting and reporting experiments and supports the gap in stating the research objectives clearly.

**Table 2.** Move 1 Step 1: Reference to the research purposes of experiment is exemplified in the following instances.

ELR 5	*To apply error-control coding techniques using Linear Block Codes.* *To design and generate Linear Block Codes with MATLAB software.*
ELR 13	*To trace out the standing wave pattern developed along a waveguide.* *To make direct SWR measurements using an SWR meter.* *To make indirect SWR measurements using the Double Minimum Method.*
ELR 27	*To construct a power measurement in DC circuit by a wattmeter.*
ELR 20	*To design and construct a prototype of a functioning programmable digital alarm clock using uP 8051.* *Apply and integrate the micro-processor & interfacing principles taught in ECP2216 course.*
ELR 35	*To study the effect of magnetic inductance of the given circuit.* *To measure self-inductance and mutual inductance.*
ELR 17	*To examine a message signal, a carrier signal and an AM modulated waveform in time domain.* *To measure the modulation index of an AM signal.* *To examine a message signal, a carrier signal and an AM modulated waveform in frequency domain.*
ELR 30	*In this experiment we will examine a message signal, a carrier signal and a FM modulated waveform in the time domain.* *To examine a message signal, a carrier signal and a FM modulated waveform in frequency domain.* *And later demonstrate the demodulation of an FM signal.*

**Move 1 Step 2: Giving an overview of the topic under study**

Move 1 Step 2 provides an overview of the topic under study. It gives general information about what the experiment is all about, states the characteristics of the variables under study, the main terms used in this experiment, short definition of the functions of each variable and features used that gives an overall view of the experiment conducted. This step has occurred in 33 reports or 94% of the total report (conventional) and but was in correct sequence in only 9 reports as step 2. This shows that the move pattern in introduction section is not always in sequence of M1S1-M1S2-M1S3-M1S4.

**Table 3.** Move 1 Step 2: Giving an overview of the topic under study is exemplified in the following instances.

ELR 22	*SWR stands for standing wave ratio or VSWR stands for Voltage standing wave ratio. SWR is computed from the ratio between an RF signal going in the forward direction (toward antenna) and the RF signal going in the reverse, or reflected direction (toward transmitter) on a transmission line. As I read the explanations given in the lab sheet i tried not to make any judgements or jump to conclusions about the meanings, A low SWR refers to a large forward RF signal and small reflected signal. Since the reflected voltage can never be less than zero the very lowest value possible is 1 or 1:1. A high SWR refers to a large reflected signal. For example, a meter reading of 9.5 indicates an SWR of 9.5 to 1.*
ELR 18	*Pulse code modulation (PCM) is essentially an analog-to-digital conversion (ADC) process where the information contained in the instantaneous samples of an analog signal is represented by digital codewords in a serial bit stream. This can be accomplished by representing the signal in discrete form in both time and amplitude domain.*
ELR 24	*Baseband digital signals are suitable for transmission over a pair of wires or coaxial cables due to its sizable power at low frequencies. These signals cannot be transmitted over a radio link because this would require impractically large antennas to efficiently radiate the low-frequency spectrum of the signal. Hence, for such purposes, we use analogue modulation techniques in which the digital signal messages are used to modulate a high-frequency continuous-wave (CW) carrier.*

**Move 1 Step 3: Reference to theoretical knowledge in the field**

Move 1 Step 3 provides reference to the theoretical knowledge in the field. This move provides students or readers with sufficient mathematical or theoretical background to understand how the experiment works, what has the earlier assumptions indicated and how the experiment is related to the theoretical knowledge. This section may be written in short if it can be well understood and connection can be made with the measurement of an experiment. Move 1 Step 3 occurred in only 20 reports or 57% (optional). Moreover, this step occurred as step 4 in 17 instances or 49% thus neither in accordance with university guidelines nor in correct sequence of M1S1-M1S2-M1S3 and M1S4. Based on the interview with subject specialist, these reports are lacking in referencing underpinning theories and citing previous literature thus making the report less concrete. A robust intervention is required in teaching EELR writing conventions to reinforce students’ ability to provide proper referencing to theories and literature.

**Table 4.** Move 1 Step 3: Reference to the theoretical knowledge in the field is exemplified in the following instances.

ELR 35	*When the equation R _1_R _4_ = R _2_R _3_, for the circuit of Figure 1.1 is satisfied, the bridge is balanced and a “null” or zero reading is obtained on the detector. Consider the Wheastone's bridge shown in Figure 1.1. It has four arms each having a resistance. The voltage at the nodes A and B may be computed using the simple potential division rule* $V_{A}=\frac{R_{3}}{R_{1}+R_{3}}\cdot V_{S}$
ELR 29	*An electronic filter is careful circuit that can be specified to attenuate other signal than selected band of frequency. There is two type of filter which is active and passive. Passive consist only resistor, inductor and capacitor while active filter consist of band amplification with a few frequency-selective passive components.*
ELR 16	*A radio frequency signal can be sent between two antennas at transmitting and receiver station. Besides, bandwidth used to transmit an AM signal usually allocate in between 200 kHz to 25MHz.*
ELR 34	*In extrinsic materials, investigation of the conduction properties gives information about the majority carriers; in intrinsic materials, we obtain information about the combined effects of conduction electrons and holes. The conductivity, σ, is defined from the equation J = σE and, in terms of the charge carrier concentrations and mobility.*
ELR 9	*The Hall effect is based on a moving charge that experience a Lorentz force in a magnetic field of F = q υ x B (= qυ _x_B _z_). Consider a n-type semiconductor (germanium), the Lorentz force due to B _z(magnetic field)_ will exert an average downward force on the electron flowing in the negative x-direction (as show as the Figure 1).*

Move 1 Step 4: Stating the main apparatus used to conduct the experiment occurred in the introduction section of 33 ELR’s or 94% (conventional) but 29 reports or 83% of the total reports just stated or outlined the main apparatus used without detailed explanation on how to use it. This is another lack identified that most of the ELRs did not provide detailed description of the apparatus used. Moreover, this step has occurred as step 4 in only 17 ELR’s or 49% only. This shows lack of compliance among undergraduates reporting ELR’s in sequential orders outlined by the university. Based on interview with lab instructors, they commented this step is often overlooked by EE students and suggested further reinforcement in instruction.

**Table 5.** Move 1 Step 4: Identification of main research apparatus is exemplified in the following instances.

ELR 24	*Software required is MATLAB*
ELR 25	*“Circuit Theory” experiment board* *DC Power Supply* *Dual-trace Oscilloscope* *Function Generator* *Digital Multimeter* *Connecting wires*
ELR 22	*A specialized linear-beam vacuum tube (evacuated electron tube). Klystrons are used as amplifiers at microwave and radio frequencies to produce both low-power reference signals for super heterodyne radar receivers and to produce high-power carrier waves for communications and the driving force for modern particle accelerators.*
ELR 3	*The frequency (ƒ) can be measured either using the mathematical relationship of speed of light in the waveguide medium, or rather using size resonant cavity (Cavity Wave meter). The resonant cavity wave takes circular or rectangular ship with both sides of wave guide short circuited.*

### Combination of move patterns in Introduction section.

In this section, the analysis of ELR’s focuses to determine the sequence of move and steps used to begin and end the introduction section. As noted in previous analysis, the length of each step varies with Move 1 Step 1 that shows the objectives stated are experiment written the shortest of all 4 steps. In this step, students use action verbs to state the objectives without detailed elaboration. This step clearly states the aims to be achieved by the end of experiment. 24 ELR’s or 69% of the reports began with statement of objectives. Although most of the reports start with M1S1, this finding cannot be generalized to overall EELR’s as some start from M1S1. 7 ELR’s or 20% of the overall reports starts with M1S2, 2 ELR’s or less than 6% of the reports start with M1S3 and only 1 report or 3% starts from M1S4 and 17 ELR’s or 49% of the total reports end with M1S1. This is the last step in presenting background information of the experiment. This step is frequently adopted to end the introduction section and before moving to method section. Next, 9 ELR’s or 26% of the reports end with M1 S3, while 6 ELR’s or 17% of the reports end with M1S2 and only 3 report ends with M1S1. Based on the occurrence of each step-in move 1 of introduction section and all three steps identified and discussed above are viewed as mandatory and conventional and optional steps in introduction section. The model proposed in Table 1 below encapsulates the Move and Steps made by undergraduate students of Electrical Engineering in writing their laboratory reports. As a whole, the findings are compared against the guidelines set by the university to determine the mismatch or lacks that shows minimal discussion on the theoretical aspects leading to experiment and the absence of referencing and citations to published literature, thus hinders the targeted reader who may not have prior knowledge to orientate into the introduction section of ELR. The combination of move and steps show most of the steps overlaps against each other and not in sequential order. This may pose challenges to laboratory instructors and supervisors to comprehend and assess the reports with clarity as only moderate level of compliance shown by undergraduates to write the reports in the format and conventions outlined by the university.

**Table 6.** Model for Introduction section of ELR.

**Move 1**	**Presenting background information (Obligatory)**
by Move 1 Step 1	Reference to research purpose (Obligatory)
by Move 1 Step 2	Providing an overview of the study (Conventional)
by Move 1 Step 3	Reference to theoretical knowledge in the field (Optional)
by Move 1 Step 4	Identification of main research apparatus (Conventional)

## Conclusion

It is advised that discipline-specific engineers and English for Academic Purposes (EAP) practitioners, such as English writing instructors, work together in response to these findings. Through this partnership, the unique learning needs of engineering students can be identified, and genre-based writing training can be effectively tailored to meet these requirements. Students can improve the quality and clarity of their lab reports as well as better fulfil the requirements stated in the university guidelines by doing this. The investigation found substantial variances between the final report deliverables produced by students and the guidelines set by the university. The absence of pertinent literature evaluations and background theories is one of the most noticeable differences. This shortcoming raises questions about the theoretical underpinnings of laboratory tests. The lack of references to past studies or attempts at replication raises the possibility that undergraduates are handling laboratory experiments quite independently. The experiments may not be as compelling or have a broad application if they lack a theoretical foundation. There appears to be a lack of information provided regarding the experiment and the reasoning behind the apparatus choice. The goal of the experiment and its applicability may not be evident to readers because of this lack of background information.

Moreover, the examination revealed discrepancies from the suggested chronological sequence while writing reports. Such deviation from the recommended structure can make the evaluation procedure more difficult and compromise the reports overall cohesion and clarity. The study also underlined how crucial it is for laboratory reports to have accurate referencing and citations. The contribution of laboratory reports to the larger corpus of knowledge and literature is constrained in the absence of these academic practices. For students to place their experiments in the larger scientific discourse, it is imperative that they comprehend the need to cite prior engineering research and theories.

It is important to recognise that this study is limited to electrical engineering ELRs, and the results might not be immediately applicable to other engineering subfields. Future research should consider data triangulation from other sources, including expert validation and interviews, to provide a more thorough knowledge of how various discourse groups approach the drafting of laboratory reports. In the long run, this comprehensive study strategy can deepen our awareness of the reasons for the adoption of writing styles and norms by discourse groups and improve our comprehension of literacy processes in engineering.

## Data availability

Figshare. Genre Analysis of introduction section in electrical engineering undergraduate laboratory reports. DOI: https://doi.org/10.6084/m9.figshare.14881911.v3. ^37^

Data are available under the terms of the Creative Commons Zero “No rights reserved” data waiver (CC BY 4.0 Public domain dedication).
