# Supplementary material for: Genre analysis of introduction section in electrical engineering undergraduate laboratory reports
Source: F1000Res. 2024 Mar 22;11:163. Originally published 2022 Feb 9. [Version 3] doi: 10.12688/f1000research.73461.3 (PMC11065396; doi:10.12688/f1000research.73461.3)
Supplement: Peer review history

# Reviewer report: Reviewer response for version 3

**DOI:** 10.5256/f1000research.163629.r258425

**Published:** 2024-04-26

**Authors:** Ilyana Jalaluddin

**Affiliations:** 1 Department of Foreign Languages, Faculty of Modern Languages and Communication, Universiti Putra Malaysia, Serdang, Malaysia

No further comments

Is the work clearly and accurately presented and does it cite the current literature?

Yes

If applicable, is the statistical analysis and its interpretation appropriate?

Not applicable

Are all the source data underlying the results available to ensure full reproducibility?

Yes

Is the study design appropriate and is the work technically sound?

Yes

Are the conclusions drawn adequately supported by the results?

Partly

Are sufficient details of methods and analysis provided to allow replication by others?

Yes

Reviewer Expertise:

ESL Writing, Literacy and Technology

I confirm that I have read this submission and believe that I have an appropriate level of expertise to confirm that it is of an acceptable scientific standard.

# Reviewer report: Reviewer response for version 3

**DOI:** 10.5256/f1000research.163629.r258427

**Published:** 2024-04-16

**Authors:** Enrique Sologuren Insúa

**Affiliations:** 1 Universidad de los Andes, Las Condes, Santiago Metropolitan Region, Chile

Revisions are appropriate. I have no further comments on the article. The observations for improvement have been incorporated.

Is the work clearly and accurately presented and does it cite the current literature?

Yes

If applicable, is the statistical analysis and its interpretation appropriate?

Yes

Are all the source data underlying the results available to ensure full reproducibility?

Yes

Is the study design appropriate and is the work technically sound?

Yes

Are the conclusions drawn adequately supported by the results?

Partly

Are sufficient details of methods and analysis provided to allow replication by others?

Partly

Reviewer Expertise:

Applied linguistics; Genre Analysis; Literacy in specialised contexts; Academic and professional writing; Writing in STEM areas.

I confirm that I have read this submission and believe that I have an appropriate level of expertise to confirm that it is of an acceptable scientific standard.

# Reviewer report: Reviewer response for version 2

**DOI:** 10.5256/f1000research.146353.r184280

**Published:** 2023-08-09

**Authors:** Enrique Sologuren Insúa

**Affiliations:** 1 Universidad de los Andes, Las Condes, Santiago Metropolitan Region, Chile

The article is clear and comprehensible. It uses a theoretical and methodological framework appropriate to the nature of the object of study.

 The specialized bibliography used is complete and sufficient.

 The study follows a firmly established tradition in genre theory and provides new knowledge about the discursive rhetorical characteristics of the texts and the difficulties that students manifest in the texture and writing of the text.

**The methodology** needs to be reinforced. It is necessary to indicate the total number of words in the corpus.

 There is also a lack of data on the **statistical analytical procedures,** i.e. how the data was analyzed to obtain the respective percentages: was a statistical test used, was only descriptive statistics used? The quantitative description procedures need to be made clearer.

 In the conclusion the difficulties are presented, but they are not framed within a broader discussion of **literacy processes in engineering.** In that sense, it is suggested to add a more robust discussion component, either in the results section or in the conclusion section.

Is the work clearly and accurately presented and does it cite the current literature?

Yes

If applicable, is the statistical analysis and its interpretation appropriate?

Yes

Are all the source data underlying the results available to ensure full reproducibility?

Yes

Is the study design appropriate and is the work technically sound?

Yes

Are the conclusions drawn adequately supported by the results?

Partly

Are sufficient details of methods and analysis provided to allow replication by others?

Partly

Reviewer Expertise:

Applied linguistics; Genre Analysis; Literacy in specialised contexts; Academic and professional writing; Writing in STEM areas.

I confirm that I have read this submission and believe that I have an appropriate level of expertise to confirm that it is of an acceptable scientific standard, however I have significant reservations, as outlined above.

# Response

**Published:** 2024-03-11

**Authors:** Veeramuthu Veerappan

**Affiliations:** Multimedia University, Malaysia

**Reviewer 2**

**1. The methodology needs to be reinforced. It is necessary to indicate the total number of words in the corpus.**

 The corpus consisting N==35 was converted and compiled into pdf and the number of words obtained are 47194.

**2. There is also a lack of data on the statistical analytical procedures, i.e. how the data was analyzed to obtain the respective percentages: was a statistical test used, was only descriptive statistics used? The quantitative description procedures need to be made clearer.**

 This study is qualitatively done. A genre analysis was conducted to determine the moves and steps that occur in the introduction section by adapting the categories outlined by Ref. 1 framework. Prior to using Ngowu’s model, a pilot test was conducted to examine the suitability of this model to the current study on introduction section of EELR. 15 EELRs were selected and examined to determine the match in the description. There were more than 60% match in the categories outlined by the model and EELRs under examination, making it the most suitable analytical framework that can be modified and replicated. The total sample size collected was N=74. These samples were further scrutinized and minimized to select the EELRs that contain the most complete and comprehensive information. Quality of data, amount of information, nature of topic, scope of study, design and method used in a qualitative study are the few factors used in determining sample size. ^9^ To control the variable, only EELRs that received distinction grades of 4 and above score over maximum score of 5 awarded by lab instructors were selected to maximise the possibility that it conforms to EE disciplinary standards.The sample size of this study is maintained at N=35 as these samples best represented the EE laboratory genre and reached a saturation level with similar recurring moves and steps. To further validate the data, a semi-structured interview was conducted with an engineering content specialist. According to the subject specialist, the total intake of electrical engineering students in this tertiary institution is not more than 100 students a year and, in each trimester, these students are engaged in writing at least 4 EELRs. [27] stated that the sample size for a qualitative study is influenced by several factors, including the quality of data, scope of the study, nature of topic, information richness from each subject, the qualitative methodology utilized, and the study's design. Thus, the data set of N=35 represents the final year EE student population of this institution. The procedures for conducting move analysis in this study adapted a corpus-based model outlined by Ref. 2 BCU approach. This approach adopted (18) and followed the procedures to determine the rhetorical function and meaning of each move and step of each text, conduct a pilot-coding to test and fine-tune the definitions of move purposes manually by hand, develop a coding protocol with clear definitions and examples of move types and steps, code the full set of texts, with an inter-rater reliability check to confirm a clear understanding of move definitions and realization of moves/steps, revise the coding protocol to resolve any discrepancies revealed by the inter-rater reliability check or newly discovered' moves/steps, and re-coding problematic areas, conduct analysis on move features and  other corpus-facilitated analyses and finally describe the corpus of texts in terms of obligatory, conventional or optional move structures.

**3. In the conclusion the difficulties are presented, but they are not framed within a broader discussion of literacy processes in engineering. In that sense, it is suggested to add a more robust discussion component, either in the results section or in the conclusion section.**

 It is advised that discipline-specific engineers and English for Academic Purposes (EAP) practitioners, such as English writing instructors, work together in response to these findings. Through this partnership, the unique learning needs of engineering students can be identified, and genre-based writing training can be effectively tailored to meet these requirements. Students can improve the quality and clarity of their lab reports as well as better fulfil the requirements stated in the university guidelines by doing this. The investigation found substantial variances between the final report deliverables produced by students and the guidelines set by the university. The absence of pertinent literature evaluations and background theories is one of the most noticeable differences. This shortcoming raises questions about the theoretical underpinnings of laboratory tests. The lack of references to past studies or attempts at replication raises the possibility that undergraduates are handling laboratory experiments quite independently. The experiments may not be as compelling or have a broad application if they lack a theoretical foundation. There appears to be a lack of information provided regarding the experiment and the reasoning behind the apparatus choice. The goal of the experiment and its applicability may not be evident to readers because of this lack of background information.

 Moreover, the examination revealed discrepancies from the suggested chronological sequence while writing reports. Such deviation from the recommended structure can make the evaluation procedure more difficult and compromise the reports overall cohesion and clarity. The study also underlined how crucial it is for laboratory reports to have accurate referencing and citations. The contribution of laboratory reports to the larger corpus of knowledge and literature is constrained in the absence of these academic practices. For students to place their experiments in the larger scientific discourse, it is imperative that they comprehend the need to cite prior engineering research and theories. It is important to recognise that this study is limited to electrical engineering ELRs, and the results might not be immediately applicable to other engineering subfields. Future research should consider data triangulation from other sources, including expert validation and interviews, to provide a more thorough knowledge of how various discourse groups approach the drafting of laboratory reports. In the long run, this comprehensive study strategy can deepen our awareness of the reasons for the adoption of writing styles and norms by particular discourse groups and improve our comprehension of literacy processes in engineering.



# Reviewer report: Reviewer response for version 2

**DOI:** 10.5256/f1000research.146353.r184286

**Published:** 2023-07-25

**Authors:** Thi Ngoc Phuong Le

**Affiliations:** 1 University of Foreign Language Studies - The University of Danang, Đà Nẵng, Vietnam

The article provides a move analysis of the Introduction section of electrical engineering undergraduate laboratory reports based on a corpus of 35 reports, adopting the approach proposed by Biber, Connor, and Upton (2007). 

 The research findings show that the Introduction section of electrical engineering undergraduate laboratory reports is structured in one move and its four subsequent steps.

 The study provides a window into how the introductory section of electrical engineering laboratory reports is organised and how much its organisational patterning comply with or deviate from university guidelines.

 There are a few concerns related to the study design and the way the results are interpreted.

 It is apparent from the results presentation that the study predicate its move analysis on a pre-existing model built on university guidelines. When this is the case, such an analytical model should be provided.

 To ensure the reliability of the data, three coders were involved, including the researcher, an engineering content specialist, and a language instructor. However, it is not quite clear how the collaboration was implemented and what inter-coder reliability coefficient was used and how it was calculated.

 Extracted from the corpus should be provided to show content realisation of the move and the steps.

 Lack of this information may make it a challenge to replicate the study.

 It would be great if insights from electrical engineering instructors who marked the students' reports were incorporated into the analysis and interpretation of the results. It would be interesting to seek their comments on the mismatches between student writing and university guidelines.

 Also, reference should be made to the epistemological properties of electrical engineering to explain why the laboratory report in electrical engineering is structured that way.

 Finally, the manuscript should be proofread again to get error free.

Is the work clearly and accurately presented and does it cite the current literature?

Yes

If applicable, is the statistical analysis and its interpretation appropriate?

Partly

Are all the source data underlying the results available to ensure full reproducibility?

Partly

Is the study design appropriate and is the work technically sound?

Partly

Are the conclusions drawn adequately supported by the results?

Partly

Are sufficient details of methods and analysis provided to allow replication by others?

Partly

Reviewer Expertise:

Genre analysis; move analysis; rhetorical features; phraseology; corpus linguistics; academic writing

I confirm that I have read this submission and believe that I have an appropriate level of expertise to confirm that it is of an acceptable scientific standard, however I have significant reservations, as outlined above.

# Response

**Published:** 2024-03-11

**Authors:** Veeramuthu Veerappan

**Affiliations:** Multimedia University, Malaysia

**Comments from reviewer 1**

 Details of amendment made in the manuscript based on reviewers comments

**1. It is apparent from the results presentation that the study predicate its move analysis on a pre-existing model built on university guidelines. When this is the case, such an analytical model should be provided.**

**Revision and/or justification:**

 The coursework specification guideline was provided to students before they embark into writing ELR which was discussed and agreed with students prior to assigning the lab report task.

 Here is the guideline provided by the university:

 To provide FOE’s method writing guideline

 According to guidelines provided by Faculty of Engineering (FOE) in MMU, the introduction section should contain the following:

1. An introduction is necessary to give an overview of the overall topic and the purpose of the report.
2. The motivation to the initialization of the experimental work can be included.
3. Its content should be general enough to orientate the reader gracefully into the subject materials.

Based on the university guidelines, below are the rhetorical structures required for the introduction section.

 1.This section is to discuss the theoretical aspects leading to the experiment.

 2.Typically, this involves the historical background of the theories published in the research literature and the questions or ambiguities arose in this theoretical work.

 3.Citations for the sources of information should be given in one of the standard bibliographic formats (for example, using square brackets with the corresponding number [2] that points to the List of References).

 4.Explore this background to prepare the readers to read the main body of the report.

 5.It should contain sufficient materials to enable the readers to understand why the set of data are collected, and what are the salient features to observe in the graph, charts and tables presented in the later sections.

 6.Depending on the length and complexity of the report, the introduction and the theoretical background may be combined into one introductory section (FOE)

**2. To ensure the reliability of the data, three coders were involved, including the researcher, an engineering content specialist, and a language instructor. However, it is not quite clear how the collaboration was implemented and what inter-coder reliability coefficient was used and how it was calculated**

 As to address the trustworthiness of this study, a coding protocol was developed with definitions and examples for mandatory, conventional and optional moves and an inter-coder reliability check was conducted among three coders ., consisting firstly the researcher, secondly an engineering content specialist and thirdly a language expert to determine disagreement or discrepancies and the coding protocol was revised. A coding scheme was developed to identify rhetorical moves and steps in introduction section and also to control the variability in the analysis that guide all coders. There were three coders involved in the development and modification of the coding scheme who is the researcher himself, secondly an engineering content specialist and thirdly a language instructor. The reason for  including two other coders apart from the researcher is to ensure inter-coder reliability. The second and third coders were trained to read two similar samples of E ELR’s and identify the moves and steps in the Introduction section.

 Cohen & Kappa coefficient, a statistical measure that takes into consideration random agreement  between agreement between coders, was used to evaluate intercoder reliability. To determine the degree of agreement above and beyond what would be predicted by chance, kappa statistics were computed. An online calculator was used to calculate Kappa statistics by comparing the coded data for a subset of the EELRs from all three coders. Following their separate codes for the subset of reports, the coders got together to discuss their consensus and differences. Each coder then went on to rate nine more reports. With a coefficient of 0.91, the Kappa coefficient obtained from this analysis demonstrated a high degree of agreement. This supports the validity of the study’s conclusions and shows a high level of dependability in the coding procedure.

**3.** **Extracted from the corpus should be provided to show content realisation of the move and the steps.Lack of this information may make it a challenge to replicate the study**

 To depict how the texts are analysed, the extracts of text from EELR corpus which show moves and steps are exemplified. The move and steps are identified such as (M1S1 means Move 1 Step 1). The extracted text used for exemplification is bolded and italicised to show the differences in analysis. Example:

 Move 1

 Presenting Background Information

 Move 1 Step 1

 Reference to research purposes

 -To verify Thevenin’s Theorem.

 -To verify Superposition Theorem.

 Please refer to manuscript for detailed exemplification.

**4.** **It would be great if insights from electrical engineering instructors who marked the students' reports were incorporated into the analysis and interpretation of the results. It would be interesting to seek their comments on the mismatches between student writing and university guidelines.**

 The insights of the engineering content specialists towards students’ compliance to university report writing guidelines based on semi-structured interviews are discussed in method and result sections. The mismatches highlight the existing gaps in writing this genre.

**5.** **Also, reference should be made to the epistemological properties of electrical engineering to explain why the laboratory report in electrical engineering is structured that way.**

 11 new references on epistemological properties of electrical engineering studies studies are discussed and added:

26. Caglak, S. (2017). Does hands-on science practices make an impact on achievement in science? A meta-analysis.  *Journal of Education in Science Environment and Health*, *3*(1), 69-87

2 7. . Mertler, C. A. & Charles, C. M. (2005). *Introduction to educational research* .(5 th Edition). Boston: MA: Pearson

28. Winberg, C., Winberg, S., Jacobs, C., Garraway, J., & Engel-Hills, P. (2016). ‘I take engineering with me': epistemological transitions across an engineering curriculum.  *Teaching in Higher Education*,  *21*(4), 398-414.

29. Baillie, Caroline, and Rita Armstrong. 2013. “Crossing Knowledge Boundaries and Thresholds: Challenging the Dominant Discourses Within Engineering Education.” In Engineering Education for Social Justice: Critical Explorations and Opportunities, edited by Juan Lucena, 135–152. Dordrecht: Springer

30. Carstensen, Anna-Karin, and Jonte Bernhard. 2008. “Threshold Concepts and Keys to the Portal of Understanding: Some Examples from Electrical Engineering.” In Threshold Concepts Within the Disciplines, edited by Ray Land, Jan H. F. Meyer, and Jan Smith, 143–154. Rotterdam: Sense

31. Garrison, Lari, Daniel M. Amos, Reed Stevens, and Andy Jocuns. 2007. “Engineering as Lifestyle and a Meritocracy of Difficulty: Two Pervasive Beliefs among Engineering Students and Their Possible Effects.” Paper presented at the 2007 Annual Conference & Exposition of the American Society for Engineering Education, Honolulu, HI. https://peer.asee.org/2791.

32. McKenna, Ann, Jeffrey Froyd, C. Judson King, Thomas Litzinger, and Elaine Seymour. 2011. “The Complexities of Transforming Engineering Higher Education: Report on Forum on Characterizing the Impact and Diffusion of Transformative Engineering Education Innovations.” https://www.nae.edu/File.aspx?id=52358

33. McKenna, Ann, Jeffrey Froyd, and Thomas Litzinger. 2014. “The Complexities of Transforming Engineering Higher Education: Preparing for Next Steps.” Journal of Engineering Education 103 (2): 188–192. doi:10.1002/jee.20039

34. Beck, John, and Michael F. D. Young. 2005. “The Assault on the Professions and the Restructuring of Academic and Professional Identities: A Bernsteinian Analysis.” British Journal of Sociology of Education 26: 183–197. doi:10.1080/0142569042000294165.

35. Bernstein, Basil. 2000. Pedagogy, Symbolic Control and Identity: Theory, Research and Critique. Oxford: Rowman & Littlefield.

36. Stiwne, Elinor Edvardsson, and Thomas Jungert. 2010. “Engineering Students’ Experiences of Transition from Study to Work.” Journal of Education and Work 23 (5): 417–437. doi:10. 1080/13639080.2010.515967.

 37.  Gumaelius, L., Skogh, I. B., Matthíasdóttir, Á., & Pantzos, P. (2024). Engineering education in change. A case study on the impact of digital transformation on content and teaching methods in different engineering disciplines.  *European Journal of Engineering Education*,  *49*(1), 70-93

**6.** **Finally, the manuscript should be proofread again to get error free.**

 The manuscript is thoroughly proofread for content, language and grammar. Changes are tracked.



# Reviewer report: Reviewer response for version 1

**DOI:** 10.5256/f1000research.77113.r139566

**Published:** 2022-06-20

**Authors:** Donnie Tulud

**Affiliations:** 1 Graduate School and Department of English Language and Literature, University of Southern Mindanao, Cotabato, Philippines

This article aims to examine the genre of Engineering Laboratory Reports (ELR) introduction section written by Electrical Engineering Undergraduates in a higher learning institution. The aims of this study are to identify the rhetorical moves and combinations of move patterns used by engineering students to write the introduction section of ELR. However, there are several issues that I want to clarify specifically with the following parts:

**Introduction**

 Though the writer has presented the problems well, still; this part lacks related concepts and studies that could substantiate the current study so as to establish the research gap. In here, the writer can provide pieces of information to make the study more problematic. Thus, it is necessary for the writer to use other studies as reference that have explored other research introduction as corpora.

**Methods**

 Why did you not consider the framework of JOHN SWALES which is the CARS model in analyzing the introduction, since you made mention of CARS model in your introduction? I believe the CARS model in describing introduction is much more comprehensive than Ngowu. Swale's model should also be considered because his framework might also generate a more comprehensive result. It can also be a confluence of these two models to see if the two have similar or different move or steps in presenting the research introduction. 

**Results**

 The meat of determining move structure is through identifying linguistic markers that realize the move or steps. Therefore, in presenting the results, the writer should include salient linguistic markers that realize each step and move. Also, the writer, should also get the frequency of each move and step to determine what move is obligatory, optional, non-evident and conventional. By doing such, the reader will know the most common moves being used and neglected in writing research introduction. Moreover, it is recommended to provide samples from the corpora on how these moves and steps are realized and presented in Engineering Laboratory Reports (ELR) introduction section written by Electrical Engineering Undergraduates

 Overall, the research paper is excellent in terms of presentation and overall findings. However, there is just a need to add more literature and related studies to give substance to the problem and establish the gap. As mentioned, there is a need to add linguistic features to the analyses so that it would be easier for the readers to identify characteristics of each move and step.

Is the work clearly and accurately presented and does it cite the current literature?

Partly

If applicable, is the statistical analysis and its interpretation appropriate?

Not applicable

Are all the source data underlying the results available to ensure full reproducibility?

Yes

Is the study design appropriate and is the work technically sound?

Yes

Are the conclusions drawn adequately supported by the results?

Yes

Are sufficient details of methods and analysis provided to allow replication by others?

Yes

Reviewer Expertise:

Genre analysis and discourse analysis

I confirm that I have read this submission and believe that I have an appropriate level of expertise to confirm that it is of an acceptable scientific standard, however I have significant reservations, as outlined above.

# Response

**Published:** 2023-03-30

**Authors:** Veeramuthu Veerappan

**Affiliations:** Multimedia University, Malaysia

Dear Dr Donnie,

 Thank you for the feedback provided. Below are our revision based on each comments given.

**List of Amendments  :**

 Details of amendment made in the manuscript. Only a paraphrased and simplified version of the revision was added in the manuscript. The full text was not reported in the manuscript due to word limitation **.**

**Introduction**

Reviewer 2

 The writer can provide pieces of information to make the study more problematic. Thus, it is necessary for the writer to use other studies as reference that have explored other research introduction as corpora.

 Revision with justification:

 There are several related concepts and studies that could substantiate the current study and establish the research gap. Analysis of genre in academic writing has received a lot of attention (Swales, 1990; Bhatia, 1993; Hyland, 2000). Finding the norms, expectations, and communication goals of certain discourse groups is the task of genre analysis (Swales, 1990), and offers a theoretical framework to analyze the genre of ELR introductions. Writing skills in engineering education has been the focus of studies, and it has been determined that engineering students have difficulty writing clearly (Larson & Murray, 2008; Lunsford, 2012). Notable studies (Swales, 1990; Hyland, 2000; Samraj, 2002) have been conducted on the moves and writing styles employed in academic writing. The concept and framework bridge the research gap for all the reason stated above, making this study significant and relevant.

 There may be a few causes for the recent lack of attention paid to this subject. Research in the field of engineering education has switched its attention to other areas, namely the use of technology in teaching and learning, assessment procedures, or diversity and inclusion in engineering classrooms. Moreover, the material already available on ELR introduction sections may be deemed sufficient and complete. In addition, the scant attention paid to this field in recent years may also have been a result of a lack of funding or other resources for research in this field.

**Methods**

Reviewer 2

 Why did you not consider the framework of JOHN SWALES which is the CARS model in analyzing the introduction, since you made mention of CARS model in your introduction?

Justification

 This study adopted Ngowu's framework because it is better suited to meet the study's objectives and unique research problems. The Ngowu's framework has been adopted or accepted in the field of engineering education research. The intention was also to apply and test this framework for assessing the introduction section of ELRs. It's also possible that the study compared the findings after using both frameworks for their analysis, but this may be beyond the scope of this study and could be recommended for future study **.** Generally, the choice of framework depends on various criteria, including the research topic, aims, and the researcher's experience and skill with the framework. Since the research was conducted on all IMRDC sections of ELR, Ngowu (1997) framework was found to be more suitable.  

**Results**

Reviewer 2

 Therefore, in presenting the results, the writer should include salient linguistic markers that realize each step and move.

 Also, the writer, should also get the frequency of each move and step to determine what move is obligatory, optional, non-evident and conventional.

Response

 Thank you for your comments on our study. We appreciate your feedback and have carefully considered your suggestions. Regarding the lack of linguistic markers in our study, we acknowledge that these are important in analyzing the introduction section of Engineering Laboratory Reports (ELRs). However, we focused on identifying the rhetorical moves and move patterns used by engineering students in writing the introduction section of ELRs. The linguistic markers are not included as they have been published in a previous study (DOI: 10.2991/978-2-494069-61-9_24

 and https://www.atlantis-press.com/proceedings/cless-22/125980201

 We agree that combining the framework used in our study with the linguistic markers identified in previous research could provide a more comprehensive analysis of ELRs. Therefore, we plan to consider incorporating these markers in future research to enhance the analysis of ELRs. We appreciate your suggestions and feedback, and we will make sure to incorporate them into future research.

 Thank you for your feedback. We agree that providing samples from the corpora on how the moves and steps are realized in the introduction section of ELR would have been useful for the readers. We have categorised the type of moves to obligatory, conventional and optional in the revision. However, due to the limited word count of the journal, we had to prioritize providing more significant information on the study's aims, methodology, and findings. Therefore, we hope that the description of the methodology used, and the findings of the study provided in this manuscript will be sufficient for readers to understand the study's contribution to the field.

# Reviewer report: Reviewer response for version 1

**DOI:** 10.5256/f1000research.77113.r126416

**Published:** 2022-04-04

**Authors:** Ilyana Jalaluddin

**Affiliations:** 1 Department of Foreign Languages, Faculty of Modern Languages and Communication, Universiti Putra Malaysia, Serdang, Malaysia

The authors have discussed the background of the study and methodology in great detail.

 The findings manage to give an overall overview of how moves have actually been used in the engineering report. However, the findings lack discussion such as the indication of what it means if the certain steps occur less in the report. The authors only state percentage and perhaps it will be more clear if the authors provide discussion and interpretation of the findings so that readers will have better view of what it means when occurrence is low or high in the report.

 Conclusion summarises the whole thing by pointing out the strengths and weaknesses of the study. But it is not connected properly to the findings. How or in what way does it mismatch with the university guidelines? What content is lacking here? Perhaps, the authors can explain further on these two matters as it helps to elaborate more about the findings.

Is the work clearly and accurately presented and does it cite the current literature?

Yes

If applicable, is the statistical analysis and its interpretation appropriate?

Not applicable

Are all the source data underlying the results available to ensure full reproducibility?

Yes

Is the study design appropriate and is the work technically sound?

Yes

Are the conclusions drawn adequately supported by the results?

Partly

Are sufficient details of methods and analysis provided to allow replication by others?

Yes

Reviewer Expertise:

Literacy studies, ESL writing skills, Technology and literacy

I confirm that I have read this submission and believe that I have an appropriate level of expertise to confirm that it is of an acceptable scientific standard, however I have significant reservations, as outlined above.

# Response

**Published:** 2023-03-30

**Authors:** Veeramuthu Veerappan

**Affiliations:** Multimedia University, Malaysia

Dear Dr Ilyana,

 Thank you for the feedback provided. Below are our revision based on each comments. 

**List of Amendments  :**

 Details of amendment made in the manuscript: Only a paraphrased and simplified version of the revision was added in the manuscript. The full text was not reported in the manuscript due to word limitation allowed by publisher **.**

Reviewer 1

 The findings lack discussion such as the indication of what it means if the certain steps occur less in the report. Provide discussion and interpretation of the findings.  

 The following is added and paraphrased:

- The introduction section of the ELR corpus consists of one main move, which is presenting background information, and four subsequent steps: referencing research purposes, providing an overview of the subject under study, referencing theoretical knowledge in the field, and identifying the primary apparatus used to conduct the experiment. The majority of the ELRs examined featured all four processes, but the study also discovered that the sequence and frequency of these steps varied among the ELRs examined. Overall, the study's findings are highly consistent with research on biochemistry articles from earlier studies, showing that this structure is typical in the industry. Therefore, authors in the field of ELR typically start their introductions by giving a general overview of the subject and establishing the background information for their experiment. This indicates ELR writers assume their readers are already familiar with the subject and do not need to provide a thorough introduction.This would imply that ELR writers are especially interested in identifying and resolving distinct issues within their field. This study suggest that ELR writers are more concerned with presenting their own research than they are with summarising earlier work in the same topic.This may also indicate that ELR writers have a keen interest in finding and solving specific research gaps in their area of study. The specific research problem or hypothesis that they provide holds mores significance to ELR writers.When analysing these results, it's critical to keep in mind that the percentages provided only reflect the frequency of each move over the whole ELR corpus and may not necessarily be representative of all introductions in the field. Furthermore, it's likely that the frequency of each motion may differ based on several factors. Yet, these results offer helpful information about the general structure and relevance of introductions in the field of ELR.

Reviewer 1

1. Conclusion summarises the whole thing by pointing out the strengths and weaknesses of the study. But it is not connected properly to the findings.
2. How or in what way does it mismatch with the university guidelines? What content is lacking here?

Revision made:

1. The study's conclusion is based on several findings made during the investigation of the ELR corpus. First, the study identified discrepancies between university guidelines and students' final outputs, particularly the exclusion of underpinning theories or important literature reviews. The students require more guidance in comprehending the expectations of the genre and the significance of incorporating background information is corroborated by this study. Additionally, the study revealed that there wasn't enough content in the introduction section of the ELR corpus to give readers a clear understanding of the experiment's topic. This finding is similar to the findings of other studies that students require assistance in honing their writing skills so they can incorporate the relevant details they need to engage with their readers.

Reviewer 1

 The findings of the study also confirmed that while experiential learning is a good strategy, it might not always add to the body of knowledge in the engineering area. Collaboration between English for Academic (EAP) practitioners and subject matter experts from the engineering profession is necessary to advance genre-based writing instruction and to determine students' learning needs. Overall, the study's results call for a need for collaborative effort to enhance writing skills as well as the need to support students in comprehending the requirements of the ELR genre.

- The study's conclusion highlights various discrepancies between the recommended format for lab reports following the university’s guidelines and the actual report the students' produce. Firstly, the absence of underpinning theories or relevant literature review, which is important to convince readers that the theory guides the current study. Next, there is a lack of sufficient general information to guide and engage readers to the study's purpose and need. These results are consistent with the Faculty’s report guidelines that the introduction section should give a general overview of the subject and the report's purpose and that its content should be sufficiently general to allow readers to easily engage and comprehend the contents of the report.

          Hence, the findings specifically found these two discrepancies:

- The findings claim that that students frequently neglected to mention the background theories or relevant literature in the introduction section which is crucial because it can be replicated, reproduced, and reapplied to the current study.  It is also important to convince the readers that the theory is still valid to be used for their investigation.
- Study findings also revealed that the introductions lacked the necessary general information to guide readers through the project's subject matter and provided readers with little information to comprehend the relevance and context of the study.
