# Supplementary material for: Genre analysis of introduction section in electrical engineering undergraduate laboratory reports
Source: F1000Res. 2023 Apr 27;11:163. Originally published 2022 Feb 9. [Version 2] doi: 10.12688/f1000research.73461.2 (PMC11065396; doi:10.12688/f1000research.73461.2)
Supplement: Peer review history

# Response

**Published:** 2024-03-11

**Authors:** Veeramuthu Veerappan

**Affiliations:** Multimedia University, Malaysia

**Reviewer 2**

**1. The methodology needs to be reinforced. It is necessary to indicate the total number of words in the corpus.**

 The corpus consisting N==35 was converted and compiled into pdf and the number of words obtained are 47194.

**2. There is also a lack of data on the statistical analytical procedures, i.e. how the data was analyzed to obtain the respective percentages: was a statistical test used, was only descriptive statistics used? The quantitative description procedures need to be made clearer.**

 This study is qualitatively done. A genre analysis was conducted to determine the moves and steps that occur in the introduction section by adapting the categories outlined by Ref. 1 framework. Prior to using Ngowu’s model, a pilot test was conducted to examine the suitability of this model to the current study on introduction section of EELR. 15 EELRs were selected and examined to determine the match in the description. There were more than 60% match in the categories outlined by the model and EELRs under examination, making it the most suitable analytical framework that can be modified and replicated. The total sample size collected was N=74. These samples were further scrutinized and minimized to select the EELRs that contain the most complete and comprehensive information. Quality of data, amount of information, nature of topic, scope of study, design and method used in a qualitative study are the few factors used in determining sample size. ^9^ To control the variable, only EELRs that received distinction grades of 4 and above score over maximum score of 5 awarded by lab instructors were selected to maximise the possibility that it conforms to EE disciplinary standards.The sample size of this study is maintained at N=35 as these samples best represented the EE laboratory genre and reached a saturation level with similar recurring moves and steps. To further validate the data, a semi-structured interview was conducted with an engineering content specialist. According to the subject specialist, the total intake of electrical engineering students in this tertiary institution is not more than 100 students a year and, in each trimester, these students are engaged in writing at least 4 EELRs. [27] stated that the sample size for a qualitative study is influenced by several factors, including the quality of data, scope of the study, nature of topic, information richness from each subject, the qualitative methodology utilized, and the study's design. Thus, the data set of N=35 represents the final year EE student population of this institution. The procedures for conducting move analysis in this study adapted a corpus-based model outlined by Ref. 2 BCU approach. This approach adopted (18) and followed the procedures to determine the rhetorical function and meaning of each move and step of each text, conduct a pilot-coding to test and fine-tune the definitions of move purposes manually by hand, develop a coding protocol with clear definitions and examples of move types and steps, code the full set of texts, with an inter-rater reliability check to confirm a clear understanding of move definitions and realization of moves/steps, revise the coding protocol to resolve any discrepancies revealed by the inter-rater reliability check or newly discovered' moves/steps, and re-coding problematic areas, conduct analysis on move features and  other corpus-facilitated analyses and finally describe the corpus of texts in terms of obligatory, conventional or optional move structures.

**3. In the conclusion the difficulties are presented, but they are not framed within a broader discussion of literacy processes in engineering. In that sense, it is suggested to add a more robust discussion component, either in the results section or in the conclusion section.**

 It is advised that discipline-specific engineers and English for Academic Purposes (EAP) practitioners, such as English writing instructors, work together in response to these findings. Through this partnership, the unique learning needs of engineering students can be identified, and genre-based writing training can be effectively tailored to meet these requirements. Students can improve the quality and clarity of their lab reports as well as better fulfil the requirements stated in the university guidelines by doing this. The investigation found substantial variances between the final report deliverables produced by students and the guidelines set by the university. The absence of pertinent literature evaluations and background theories is one of the most noticeable differences. This shortcoming raises questions about the theoretical underpinnings of laboratory tests. The lack of references to past studies or attempts at replication raises the possibility that undergraduates are handling laboratory experiments quite independently. The experiments may not be as compelling or have a broad application if they lack a theoretical foundation. There appears to be a lack of information provided regarding the experiment and the reasoning behind the apparatus choice. The goal of the experiment and its applicability may not be evident to readers because of this lack of background information.

 Moreover, the examination revealed discrepancies from the suggested chronological sequence while writing reports. Such deviation from the recommended structure can make the evaluation procedure more difficult and compromise the reports overall cohesion and clarity. The study also underlined how crucial it is for laboratory reports to have accurate referencing and citations. The contribution of laboratory reports to the larger corpus of knowledge and literature is constrained in the absence of these academic practices. For students to place their experiments in the larger scientific discourse, it is imperative that they comprehend the need to cite prior engineering research and theories. It is important to recognise that this study is limited to electrical engineering ELRs, and the results might not be immediately applicable to other engineering subfields. Future research should consider data triangulation from other sources, including expert validation and interviews, to provide a more thorough knowledge of how various discourse groups approach the drafting of laboratory reports. In the long run, this comprehensive study strategy can deepen our awareness of the reasons for the adoption of writing styles and norms by particular discourse groups and improve our comprehension of literacy processes in engineering.

                                                                      

# Response

**Published:** 2024-03-11

**Authors:** Veeramuthu Veerappan

**Affiliations:** Multimedia University, Malaysia

**Comments from reviewer 1**

 Details of amendment made in the manuscript based on reviewers comments

**1. It is apparent from the results presentation that the study predicate its move analysis on a pre-existing model built on university guidelines. When this is the case, such an analytical model should be provided.**

**Revision and/or justification:**

 The coursework specification guideline was provided to students before they embark into writing ELR which was discussed and agreed with students prior to assigning the lab report task.

 Here is the guideline provided by the university:

 To provide FOE’s method writing guideline

 According to guidelines provided by Faculty of Engineering (FOE) in MMU, the introduction section should contain the following:

1. An introduction is necessary to give an overview of the overall topic and the purpose of the report.
2. The motivation to the initialization of the experimental work can be included.
3. Its content should be general enough to orientate the reader gracefully into the subject materials.

Based on the university guidelines, below are the rhetorical structures required for the introduction section.

 1.This section is to discuss the theoretical aspects leading to the experiment.

 2.Typically, this involves the historical background of the theories published in the research literature and the questions or ambiguities arose in this theoretical work.

 3.Citations for the sources of information should be given in one of the standard bibliographic formats (for example, using square brackets with the corresponding number [2] that points to the List of References).

 4.Explore this background to prepare the readers to read the main body of the report.

 5.It should contain sufficient materials to enable the readers to understand why the set of data are collected, and what are the salient features to observe in the graph, charts and tables presented in the later sections.

 6.Depending on the length and complexity of the report, the introduction and the theoretical background may be combined into one introductory section (FOE)

**2. To ensure the reliability of the data, three coders were involved, including the researcher, an engineering content specialist, and a language instructor. However, it is not quite clear how the collaboration was implemented and what inter-coder reliability coefficient was used and how it was calculated**

 As to address the trustworthiness of this study, a coding protocol was developed with definitions and examples for mandatory, conventional and optional moves and an inter-coder reliability check was conducted among three coders ., consisting firstly the researcher, secondly an engineering content specialist and thirdly a language expert to determine disagreement or discrepancies and the coding protocol was revised. A coding scheme was developed to identify rhetorical moves and steps in introduction section and also to control the variability in the analysis that guide all coders. There were three coders involved in the development and modification of the coding scheme who is the researcher himself, secondly an engineering content specialist and thirdly a language instructor. The reason for including two other coders apart from the researcher is to ensure inter-coder reliability. The second and third coders were trained to read two similar samples of E ELR’s and identify the moves and steps in the Introduction section.

 Cohen & Kappa coefficient, a statistical measure that takes into consideration random agreement  between agreement between coders, was used to evaluate intercoder reliability. To determine the degree of agreement above and beyond what would be predicted by chance, kappa statistics were computed. An online calculator was used to calculate Kappa statistics by comparing the coded data for a subset of the EELRs from all three coders. Following their separate codes for the subset of reports, the coders got together to discuss their consensus and differences. Each coder then went on to rate nine more reports. With a coefficient of 0.91, the Kappa coefficient obtained from this analysis demonstrated a high degree of agreement. This supports the validity of the study’s conclusions and shows a high level of dependability in the coding procedure.

**3.** **Extracted from the corpus should be provided to show content realisation of the move and the steps.Lack of this information may make it a challenge to replicate the study**

 To depict how the texts are analysed, the extracts of text from EELR corpus which show moves and steps are exemplified. The move and steps are identified such as (M1S1 means Move 1 Step 1). The extracted text used for exemplification is bolded and italicised to show the differences in analysis. Example:

 Move 1

 Presenting Background Information

 Move 1 Step 1

 Reference to research purposes

 -To verify Thevenin’s Theorem.

 -To verify Superposition Theorem.

 Please refer to manuscript for detailed exemplification.

**4.** **It would be great if insights from electrical engineering instructors who marked the students' reports were incorporated into the analysis and interpretation of the results. It would be interesting to seek their comments on the mismatches between student writing and university guidelines.**

 The insights of the engineering content specialists towards students’ compliance to university report writing guidelines based on semi-structured interviews are discussed in method and result sections. The mismatches highlight the existing gaps in writing this genre.

**5.** **Also, reference should be made to the epistemological properties of electrical engineering to explain why the laboratory report in electrical engineering is structured that way.**

 11 new references on epistemological properties of electrical engineering studies studies are discussed and added:

26. Caglak, S. (2017). Does hands-on science practices make an impact on achievement in science? A meta-analysis. *Journal of Education in Science Environment and Health*, *3*(1), 69-87

2 7. . Mertler, C. A. & Charles, C. M. (2005). *Introduction to educational research* .(5 th Edition). Boston: MA: Pearson

28. Winberg, C., Winberg, S., Jacobs, C., Garraway, J., & Engel-Hills, P. (2016). ‘I take engineering with me': epistemological transitions across an engineering curriculum. *Teaching in Higher Education*, *21*(4), 398-414.

29. Baillie, Caroline, and Rita Armstrong. 2013. “Crossing Knowledge Boundaries and Thresholds: Challenging the Dominant Discourses Within Engineering Education.” In Engineering Education for Social Justice: Critical Explorations and Opportunities, edited by Juan Lucena, 135–152. Dordrecht: Springer

30. Carstensen, Anna-Karin, and Jonte Bernhard. 2008. “Threshold Concepts and Keys to the Portal of Understanding: Some Examples from Electrical Engineering.” In Threshold Concepts Within the Disciplines, edited by Ray Land, Jan H. F. Meyer, and Jan Smith, 143–154. Rotterdam: Sense

31. Garrison, Lari, Daniel M. Amos, Reed Stevens, and Andy Jocuns. 2007. “Engineering as Lifestyle and a Meritocracy of Difficulty: Two Pervasive Beliefs among Engineering Students and Their Possible Effects.” Paper presented at the 2007 Annual Conference & Exposition of the American Society for Engineering Education, Honolulu, HI. https://peer.asee.org/2791.

32. McKenna, Ann, Jeffrey Froyd, C. Judson King, Thomas Litzinger, and Elaine Seymour. 2011. “The Complexities of Transforming Engineering Higher Education: Report on Forum on Characterizing the Impact and Diffusion of Transformative Engineering Education Innovations.” https://www.nae.edu/File.aspx?id=52358

33. McKenna, Ann, Jeffrey Froyd, and Thomas Litzinger. 2014. “The Complexities of Transforming Engineering Higher Education: Preparing for Next Steps.” Journal of Engineering Education 103 (2): 188–192. doi:10.1002/jee.20039

34. Beck, John, and Michael F. D. Young. 2005. “The Assault on the Professions and the Restructuring of Academic and Professional Identities: A Bernsteinian Analysis.” British Journal of Sociology of Education 26: 183–197. doi:10.1080/0142569042000294165.

35. Bernstein, Basil. 2000. Pedagogy, Symbolic Control and Identity: Theory, Research and Critique. Oxford: Rowman & Littlefield.

36. Stiwne, Elinor Edvardsson, and Thomas Jungert. 2010. “Engineering Students’ Experiences of Transition from Study to Work.” Journal of Education and Work 23 (5): 417–437. doi:10. 1080/13639080.2010.515967.

 37. Gumaelius, L., Skogh, I. B., Matthíasdóttir, Á., & Pantzos, P. (2024). Engineering education in change. A case study on the impact of digital transformation on content and teaching methods in different engineering disciplines. *European Journal of Engineering Education*, *49*(1), 70-93

**6.** **Finally, the manuscript should be proofread again to get error free.**

 The manuscript is thoroughly proofread for content, language and grammar. Changes are tracked.

                                                      
